# The African Prospective study on the Early Detection and Identification of Cardiovascular disease and Hypertension (African-PREDICT): Design, recruitment and initial examination

**DOI:** 10.1177/2047487318822354

**Published:** 2019-01-06

**Authors:** Aletta E Schutte, Philimon N Gona, Christian Delles, Aletta S Uys, Adele Burger, Catharina MC Mels, Ruan Kruger, Wayne Smith, Carla MT Fourie, Shani Botha, Leandi Lammertyn, Johannes M van Rooyen, Lebo F Gafane-Matemane, Gontse G Mokwatsi, Yolandi Breet, H Salome Kruger, Tertia van Zyl, Marlien Pieters, Lizelle Zandberg, Roan Louw, Sarah J Moss, Itumeleng P Khumalo, Hugo W Huisman

**Affiliations:** 1 Hypertension in Africa Research Team (HART), North-West University, South Africa; 2 South African Medical Research Council: Unit for Hypertension and Cardiovascular Disease, North-West University, South Africa; 3 Department of Exercise and Health Sciences, University of Massachusetts Boston, USA; 4 Institute of Cardiovascular and Medical Sciences (ICAMS), University of Glasgow, UK; 5 Centre of Excellence for Nutrition, North-West University, South Africa; 6 Human Metabolomics, North-West University, South Africa; 7 Physical activity, Sport and Recreation Research Focus Area, North-West University, South Africa; 8 Department of Psychology, University of the Free State, South Africa

**Keywords:** African-PREDICT, hypertension, ethnicity, race, black, biomarkers, cohort, longitudinal, young, organ damage

## Abstract

**Background:**

Globally hypertension is stabilising, but in sub-Saharan Africa the incidence of hypertension remains on an increase. Although this might be attributed to poor healthcare and ineffective antihypertensive treatment, there is a limited understanding of population and individual-specific cardiovascular pathophysiology – necessary for effective prevention and treatment strategies in Africa. As there is a lack of longitudinal studies tracking the early pathophysiological development of hypertension in black populations, the African-PREDICT study was initiated. The purpose of this paper is to describe the detailed methodology and baseline cohort profile of the study.

**Methods and results:**

From 2013 to 2017, the study included 1202 black (*N* = 606) and white (*N* = 596) men and women (aged 20–30 years) from South Africa – screened to be healthy and clinic normotensive. At baseline, and each 5-year follow-up examination, detailed measures of health behaviours, cardiovascular profile and organ damage are taken. Also, comprehensive biological sampling for the ‘omics’ and biomarkers is performed. Overall, the baseline black and white cohort presented with similar ages, clinic and 24-hour blood pressures, but black adults had lower socioeconomic status and higher central systolic blood pressure than white individuals.

**Conclusions:**

The prospective African-PREDICT study in young black and white adults will contribute to a clear understanding of early cardiovascular disease development.

## Introduction

A recent systematic review undertaken in 19.1 million participants indicated that while blood pressure (BP) has on average decreased worldwide since 1975, region-specific estimates show that the mean systolic BP actually increased in some regions, with the highest mean BPs recorded worldwide being in African countries.^1^ The World Health Organization (WHO) Study on Global Ageing and Adult Health including adults aged over 50 years corroborated this finding, reporting that South Africa had the highest prevalence of hypertension ever reported in a nationally representative survey, with nearly four in five participants presenting with hypertension.^2^

Despite calls to increase awareness and education regarding hypertension in Africa, awareness of the condition remains dismally low, with 38% in South Africa,^2^ 23% in Ghana^2^ and 14.8% in Mozambique aware of their hypertensive status.^3^ Current practices to treat hypertension in Africa are overwhelmingly ineffective, evidenced by very low control rates of 7.8% in South Africa,^2,4^ 4.1% in Ghana^2^ and 3.1% in Mozambique.^3^ These failing practices are possibly attributable to poor healthcare systems, low attained education levels, and antihypertensive treatment not being as effective in black populations as they are in white populations.^5^

Less than 100 years ago, for example, not a single patient with hypertension could be found in a hospital in Kenya.^6^ Currently, the health behaviours consequential to urbanisation reveal the susceptibility of black populations to develop hypertension rapidly. We have demonstrated this in a South African population in transition, in which nearly one in four black participants with optimal BP (≤120 and 80 mmHg) developed hypertension over 5 years.^7^

Among the reasons for the vulnerability of black individuals to develop hypertension are several social (socioeconomic status; SES)^8^ health disparities limiting access to care, experience of stress, urbanisation, acculturation)^9^ and pathophysiological reasons (salt sensitivity, suppressed renin–angiotensin system, autonomic imbalance with sympathetic overactivity).^10–12^ But there is consensus among scientists that there are limited longitudinal studies conducted in Africa,^13,14^ and there is especially a lack of knowledge on the early pathophysiological development of hypertension in young black individuals. Focusing on youth is particularly important because most studies focus on the elderly or black patients with overt cardiovascular disease. However, we have shown in young populations,^15,16^ including 6–8 year olds, that black children already show early vascular aging when compared to white children from the same schools.^17^

To address these limitations, a clear understanding of the pathophysiological development of hypertension and subclinical organ damage over time – especially in young black populations – is required. Also required is scientific data on objectively measured health behaviours such as dietary patterns, salt intake, alcohol consumption, cigarette smoking, psychological distress and physical inactivity. The African-PREDICT study was designed to address both requirements to track and monitor longitudinally the development of hypertension in healthy black individuals, aged 20–30 years, while performing a comparison with white counterparts. This will allow the identification of ethnic-specific patterns as more research has already been carried out on populations of European descent. Furthermore, the African-PREDICT study collects modern data including genomics, proteomics and metabolomics, as well as biomarkers to explore innovatively the association of early hypertension and cardiovascular outcome in bi-ethnic adults with no hypertension. These high-tech and modern biomarkers will enable precision public health,^18^ and have the potential to lead to novel and personalised strategies in preventing and treating hypertension in Africa. In this paper we aimed to describe the detailed methodology, including the design, recruitment and key baseline results of the study.

## Methods

### Design and sample size estimation

This study was designed to track and monitor the development of hypertension in a bi-ethnic sample, and thus follows an observational longitudinal study design. To estimate the sample size we used an ethnicity/age/sex/SES stratified sampling design, set to 200 black and 200 white in each of three SES categories for the 20–30-year-old age group (for a total of 600 black and 600 white participants, with equal sex distribution). The detailed sample size estimation is described in the Supplementary material.

### Study setting

**Figure 1.**
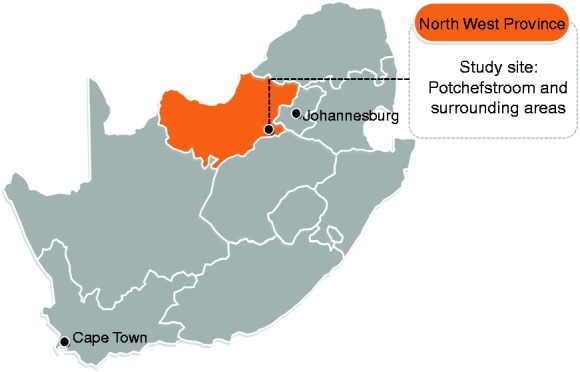
Sample collection for the African-PREDICT study took place in the city of Potchefstroom and surrounding areas within central South Africa, 2013–2017.

Participant recruitment was conducted in and around the city of Potchefstroom, in the JB Marks local municipality, North West Province in central South Africa (Figure 1). Based on census data from 2016, the population size of the municipality is 243,527, with 155,361 between the ages of 15 and 59 years. A total of 77.1% was black Africans, 16.9% white, 5.3% of mixed origin and 0.4% of Indian/Asian ethnicity. The majority of persons aged 20 years and older had secondary schooling as their highest level of education (65.1%).

### Participant recruitment and eligibility

The study recruited apparently healthy volunteers aged 20–30 years. Participants were recruited at workplaces, through public advertisements on radio, noticeboards and newspapers. The study design included recruitment of self-reported African (black) participants, and those of European (white) descent for comparison. Besides balanced selection based on race and sex, participants were also stratified by SES. SES is calculated using a point system that was adapted from Kuppuswamy's Socioeconomic Status Scale^19^ for a South African environment, scoring participants in three categories: skill level, education and household income. Scores were used to categorise according to low, middle and high socioeconomic groups.

**Figure 2.**
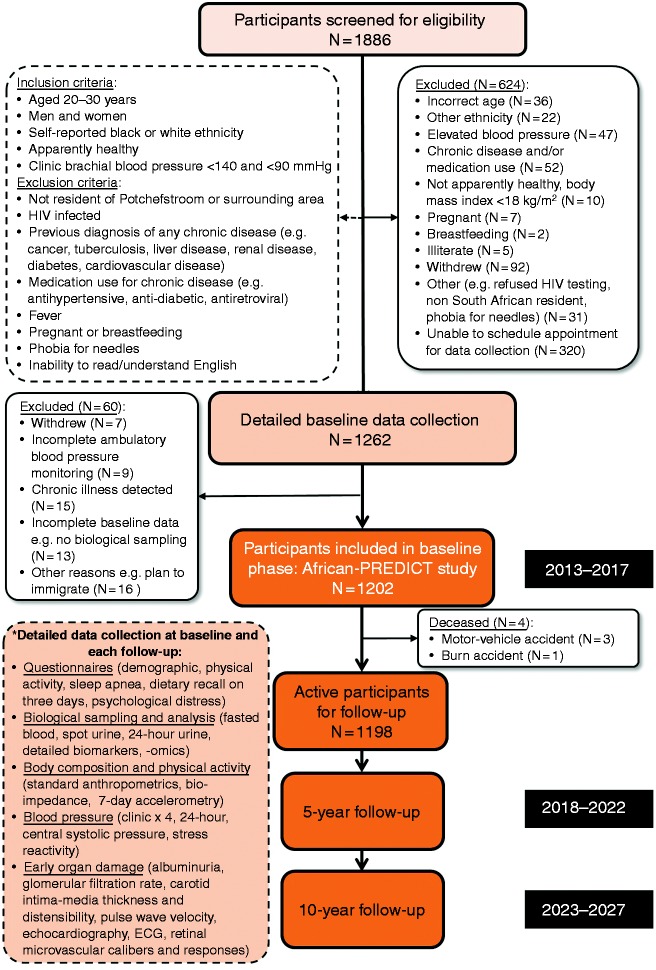
Consort diagram illustrating research participant inclusion and data collection in the African-PREDICT study, Potchefstroom, South Africa, 2013–2017.

Volunteers (*N* = 1886) underwent screening to determine eligibility. To be eligible, participants were required to have clinic brachial systolic BP less than 140 mmHg and diastolic BP less than 90 mmHg,^20^ be uninfected with HIV, have no self-reported previous diagnosis or medication use for chronic disease, not be pregnant or breastfeeding if women (Figure 2). Once eligible participants were identified through screening, they were invited to another clinic visit appointment for baseline data collection. The median time between screening and research data collection was 15 days (interquartile range 7–40 days).

Baseline exam cycle and data collection took place within the Hypertension Research and Training Clinic on the Potchefstroom campus of the North-West University between 2013 and 2017. Of the 1886 individuals who underwent screening, 1202 met eligibility criteria and were finally included (606 were black; 596 were white). Figure 2 shows a consort diagram depicting a summary of participant disposition including reasons for ineligibility. More than half (51.3%) of the 624 were ineligible because they could not schedule the baseline appointment at the clinic. Ninety-nine (15.9%) had prevalent hypertension or were taking medication for a chronic disease.

### Organisational procedures during data collection

Data collection was performed under highly controlled conditions within a hypertension clinic. The space in the clinic consists of a reception with spacious waiting area, dining area, and six temperature-controlled private assessment rooms. The per-participant assessments are extensive, the clinic therefore schedules and processes a maximum of four participants each day between the months of February and November. For the baseline exam cycle, annually the clinic processed 208 eligible participants in 2013, 260 in 2014, 219 in 2015, 220 in 2016 and 295 in 2017.

Information leaflets are provided to participants prior to the day on which the study measurements are performed. Participants are required to fast from 22:00 hours the evening before the day of the study. They are transported free of charge to the hypertension clinic, arriving at approximately 08:00 hours, where they are familiarised with the research environment and experimental set-up, and written informed consent is obtained.

Early morning spot urine samples are collected, and before 09:30 hours blood samples taken with a sterile winged infusion set and syringes from the antebrachial vein. All blood samples are immediately taken to the onsite laboratory, centrifuged to obtain plasma or serum and aliquoted into cryovials for storage in biofreezers at −80 ℃ until analysis. After blood sampling, a range of questionnaires (Table 1 and Supplementary material) are completed throughout the morning with guidance from the research staff. In parallel, anthropometry, bio-impedance and a set of cardiovascular measurements are performed (Table 1 and Supplementary material), and participants are provided with a light meal (excluding caffeine). When all measurements and assessments are completed, participants are given instructions to obtain a 24-hour urine sample, a 24-hour BP monitor is connected, as well as a combined heart rate and accelerometry device (ActiHeart) (to be worn for seven consecutive days). Direct feedback on clinical study assessments and measurement results is given individually to participants, and referrals made if required. At approximately 13:00 hours, transport is provided to all participants to return home. On subsequent days the study team collects the apparatus and urine samples, and over the course of a week dietary questionnaires are completed.

**Table 1.** Overview of data and samples collected at baseline and each follow-up for the African-PREDICT study, Potchefstroom, South Africa, 2013–2017.

***Questionnaires***
**General health and demographic questionnaire**: self-reported ethnicity, sex, date of birth, language, marital status, education, employment, income (derived socioeconomic status),^19^ tobacco use, alcohol use, self-reported health, family history of disease; medication use
**Global physical activity questionnaire (GPAQ):**^**41**^ total minutes (and metabolic equivalent minutes) of vigorous, moderate and sedentary activity recall over the past 7 days
**Psychosocial questionnaires**: Mental health continuum (short-form);^42,43^ stress overload scale;^44^ basic traits inventory (short form);^45^ patient health questionnaire;^46^ subjective vitality scale;^47,48^ meaning in life questionnaire;^49^ coping strategy indicator;^50^ general health questionnaire;^51^ new general self-efficacy scale;^52^ health-specific self-efficacy scales^53^
**24-Hour dietary recall questionnaire**:^54–56^ (completed three times: once on site and twice within 7 days)
**Salt frequency intake questionnaire**
**Berlin sleep apnea questionnaire**:^57^ snore and sleep indicators
***Body composition***
**Anthropometry**: height (SECA 213 Stadiometer, SECA, Hamburg, Germany); weight (SECA 813 Electronic scale, SECA, Hamburg, Germany); waist, hip and neck circumference (Lufkin steel anthropometric tape, W606PM; Lufkin, Apex, USA), body mass index, body surface area
**Bio-electrical impedance**: (Bodystat 1500MDD, Bodystat, Douglas, UK): body fat %, lean mass
***Habitual physical activity monitoring***
**Combined heart rate and accelerometry over a maximum of 7 consecutive days**: (ActiHeart, CamNtech Ltd., Cambridge, UK): resting, activity and total energy expenditure
***Blood pressure***
**Clinic brachial blood pressure**: (Dinamap Procare 100, GE Medical Systems, Milwaukee, USA): systolic, diastolic, heart rate
**Clinic central blood pressure and augmentation index**: (SphygmoCor XCEL, AtCor Medical Pty. Ltd., Sydney, Australia): systolic
**24-Hour brachial blood pressure and ECG monitoring**: (Card(X)plore, Meditech, Budapest, Hungary): systolic, diastolic, heart rate, day, night, dipping status, masked hypertension
***Target organ damage***
**Carotid-femoral pulse wave velocity**: (SphygmoCor XCEL, AtCor Medical Pty. Ltd., Sydney, Australia): large artery stiffness
**Carotid intima-media thickness, carotid distensibility, plaque score**: (General Electric Vivid E9, GE Vingmed Ultrasound A/S, Horten, Norway)
**Cardiovascular reactivity during stress testing**: Cold pressor test and Stroop test (Finometer, Finapres Measurement Systems, Amsterdam, The Netherlands): blood pressure, heart rate, stroke volume, total peripheral resistance, ‘Windkessel’ arterial compliance (resting, % change)
**Retinal microvascular calibre and responses to a light flicker stimulus**: (Dynamic Vessel Analyzer; Imedos, Jena, Germany): central retinal artery and vein equivalents, peak artery dilation and constriction, peak vein dilation
**Intra-ocular eye pressure**: (Tonopen Avia Tonometer CE 0120; Reichert, Munich, Germany)
**Electrocardiography 12-lead**: (Norav Medical Ltd., PC 1200, Israel)
**Echocardiography**: (General Electric Vivid E9, GE Vingmed Ultrasound A/S, Horten, Norway):^29,58^ left ventricular mass index, relative wall thickness, E/A ratio, E/é ratio, mitral valve deceleration time, LA/Ao ratio, fractional shortening, ejection fraction
**Estimated glomerular filtration rate**: chronic kidney disease epidemiology formula, CKD-EPI^59^
**Urinary albumin-to-creatinine ratio** (spot urine); **24-hour urinary albumin excretion**
***Biological sampling***
Morning fasted blood sample
Morning spot urine on two separate days
24-Hour urine sample
***Summary of specimen repository stored at −80 ℃***
Serum (16 × 500 µl, 50 × 200 µl aliquots)
NaF plasma (2 × 500 µl aliquots)
EDTA plasma (9 × 500 µl; 21 × 200 µl aliquots)
Citrated plasma (5 × 500 µl; 8 × 200 µl aliquots)
Stabilyte plasma (citrated plasma at reduced pH) (4 × 500 µl aliquots)
EDTA whole blood (4 × 500 µl aliquots)
Spot blood samples on absorbant paper Guthrie cards
Spot urine (9 × 1.5 ml; 3 × 500 µl aliquots)
24-Hour urine (4 × 1.5 ml)
Baseline EDTA buffy coat (500 µl for DNA analysis using the Maxwell automated system) EDTA buffy coat and red blood cells mix (1000 µl)
Each follow-up: EDTA whole blood (500 µl with 300 µl RNA later) EDTA buffy coat (500 µl with 300 µl RNA later)

### What has been measured?

Table 1, Table 2 and Figure 2 provide a comprehensive list of data collected and biological samples collected at baseline and at each follow-up exam cycle. These include questionnaires (e.g. medical history, social status, diet, psychosocial profile); biological samples preserved at –80 ℃ (e.g. serum, plasma, buffy coat, 24-hour urine); biomarkers (e.g. lipids, glucose, multiplex cytokines, RAS-Fingerprint, adipokines, oxidative stress, nitric oxide and coagulation markers, urinary sodium, metabolomics, proteomics); body composition; physical activity; BP (office, 24-hour, central, reactivity); target organ damage (arterial stiffness, carotid wall thickness, electrocardiography, echocardiography, retinal microvasculature, renal function). This broad range of basic and advanced measurements is performed using gold standard methods at each visit by trained research nurses, postgraduate students and academic staff. Specific methods and measurements are described in detail in the Supplementary material.

**Table 2.** Overview of analytes included as part of biomarker analyses for the African-PREDICT study, Potchefstroom, South Africa, 2013–2017.

**Biomarker laboratory analyses performed in South Africa and in international laboratories** ****
**Serum**: albumin, total protein, liver enzymes, billirubin, uric acid, apolipoprotein A and B, calcium, chloride, cholesterol (high density, low density), triglycerides, creatinine, cystatin C, C-reactive protein, interleukin-6, tumour necrosis factor-α, iron, potassium, magnesium, sodium, phosphate, selenium, urea, cotinine, renin, aldosterone, testosterone, dehydroepiandrosterone sulfate, human sex hormone-binding globulin, cortisol, N-terminal prohormone B-type natriuretic peptide, MB isoenzyme of creatinine kinase, insulin, parathyroid hormone, 25-hydroxyvitamin D, angiotensin-converting enzyme, adiponectin, leptin, glutathione reductase, total antioxidant status, reactive oxygen species (serum peroxides), myeloperoxidase, cellular adhesion molecules, p-selectin, ADAMTS13, growth differentiating factor-15, insulin-like growth factor 1, insulin-like growth factor binding protein-3, tetrahydrobiopterin, renin–angiotensin system-Fingerprint including 10 peptides**†**
**Sodium fluoride plasma**: glucose
**Citrated plasma**: Von Willbrand factor, plasma clot turbidimetry and lysis
**Stabilyte plasma**: total fibrinogen, γ′ fibrinogen, plasminogen activator inhibitor-1
**EDTA plasma**: prorenin, monocyte chemotactic protein-1, symmetric and asymmetric dimethylarginines (SDMA, ADMA),**†** arginine,**†** homoarginine,**†** 21-multiplex T-cell inflammatory cytokines**†**
**EDTA whole blood**: full blood count, blood group, glycated haemoglobin, superoxide dismutase, glutathione peroxidase, total glutathione
**Spot urine**: albumin, creatinine, chloride, sodium, potassium, uromodullin,**†** trace elements**†** (among others selenium, palladium, lithium, lead, mercury, cadmium, copper, zinc, iron, manganese), nitrite,**†** nitrate,**†** symmetric and asymmetric dimethylarginines (SDMA, ADMA),**†** dimethylamine,**†** malondialdehyde.**†** Omics include proteomics/peptidomics,**†** metabolomics
**24-Hour urine**: volume, sodium, potassium (estimated salt intake calculated), chloride, albumin, creatinine, iodine, marinobufagenin**†**
**Selected genotyping** ^†^

^†^indicates analyses performed in international laboratories.

### Follow-up examination

The African-PREDICT population exam cycles are 5 years. At each exam participants undergo comprehensive research assessments and measurements. The first post-baseline follow-up exam cycle will take place between 2018 and 2022 (Figure 2). The second follow-up exam cycle is scheduled 5 years after the first, between 2023 and 2027. Participants are contacted annually using different platforms, including email, telephone calls, mobile cellular messages, personal visits and social media.

#### Endpoints

Since the study sample consists of apparently healthy participants in their twenties prospectively followed, we anticipate few hard clinical endpoints (e.g. stroke) in the subsequent 10 years of follow-up. We therefore focused on measuring and recording surrogate endpoints such as incident hypertension, changes in cardiovascular biomarker profiles and subclinical target organ damage. However, preliminary analyses revealed that 28% of the participants were already prehypertensive (systolic BP > 120 mmHg and/or diastolic BP > 80 mmHg) and 15% presented with masked hypertension (clinic systolic BP < 140 mmHg and diastolic BP < 90 mmHg and 24-hour systolic BP ≥ 130 mmHg or diastolic BP ≥80 mmHg). We therefore anticipate that a substantial proportion will develop sustained hypertension during the first 5-year follow-up, and an even higher proportion will develop hypertension at 10-year follow-up. As secondary endpoints, the focus will also be on ‘soft’ surrogate outcomes such as intra-individual changes from baseline in cardiovascular measures, biomarker profiles and subclinical target organ damage.

### Statistical methods

Detailed statistical methodology is described in the online Supplementary material. The goal for statistical analysis was to compare cross-sectional measures between black and white ethnicities from the baseline data collection, using Statistica 13 (TIBCO Software Ltd., Tulsa, OK, USA). Two sample methods were used. Regression methods were used to account for confounding factors.

### Ethical considerations

The African-PREDICT study was endorsed by the National Department of Health and approved and sanctioned by the North West Department of Health and Health Research Ethics Committee of the North-West University, South Africa (NWU-00001-12-A1). All participants gave written informed consent, and all procedures were in adherence with good clinical practice and the Declaration of Helsinki.

The study is registered at ClinicalTrials.gov (identifier: NCT03292094), and project progress and publication updates are periodically posted on ResearchGate (https://www.researchgate.net/project/African-PREDICT-study-African-Prospective-study-on-the-Early-Detection-and-Identification-of-Cardiovascular-disease-and-Hypertension).

## Results

**Figure 3.**
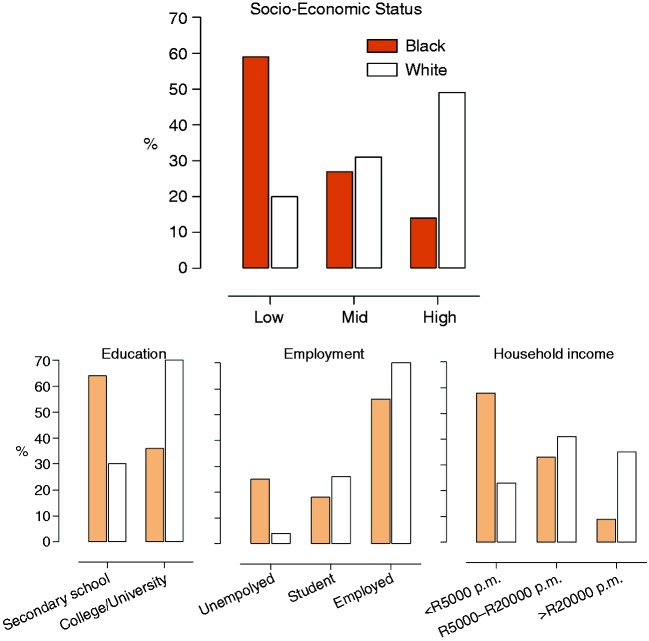
Socioeconomic status profile of black and white participants from the African-PREDICT study, Potchefstroom, South Africa, 2013–2017.

The baseline component involving 1202 eligible participants, per design was evenly split by race (606 black, 596 white) with a similar mean age (24.5 years), and even sex distribution (Table 3). Socioeconomic distribution was skewed despite significant attempts during the design and recruitment phases for balancing SES. The majority of black adults had low SES (59%), and white adults had high SES (49%), thereby reflecting the prevailing demographics in South Africa. Nevertheless, within each ethnic group there is a clear spread between low to high SES, to allow detailed analyses (Figure 3). In the following subsections some key results are highlighted – based on Table 3, but also on results published from subsets of the African-PREDICT study.

**Table 3.** Baseline profile of eligible participants of the African-PREDICT study (*N* = 1202), Potchefstroom, South Africa, 2013–2017.

	Black *N* = 606	White *N* = 596	Difference (95% CI)
Age, years	24.5	24.6	−0.11 (−0.46; 0.25)
Men/women, *N*	294/312	284/312	0.01 (−0.05; 0.06)
Socioeconomic status, *N* (%)			
Low	357 (58.9)	119 (20.0)	0.39 (0.34; 0.44)
Mid	164 (27.1)	183 (30.7)	−0.04 (−0.09; 0.01)
High	85 (14.0)	294 (49.3)	−0.35 (−0.40; −0.30)
Body composition			
Body height, cm	164	172	−8.22 (−9.19; −7.25)
Body mass index, kg/m^2^	24.6	25.5	−0.96 (−1.59; −0.33)
Waist circumference, cm	77.8	82.5	−4.73 (−6.13; −3.34)
Body fat, %	25.7	24.0	1.74 (0.56; 2.91)
Physical activity			
Activity energy expenditure, kCal	425	403	22.1 (−4.36; 48.6)
Total energy expenditure, kCal	2205	2353	−147 (−204; −91)
Estimated salt intake, g/day†	7.81	7.30	0.93 (0.98; 1.17)
Cardiovascular profile			
Clinic systolic BP, mmHg	117	116	0.98 (−0.41; 2.37)
Clinic diastolic BP, mmHg	79.4	77.7	1.67 (0.73; 2.60)
Clinic heart rate, bpm	63.9	66.7	−2.82 (−4.02; −1.62)
Clinic central systolic BP, mmHg	110	105	4.40 (3.31; 5.50)
24-Hour systolic BP, mmHg	116	118	−2.01 (−3.01; −0.94)
24-Hour diastolic BP, mmHg	68.8	68.5	0.25 (−0.42; 0.92)
24-Hour heart rate, bpm	75.2	73.6	−0.05 (−0.08; −0.01)
Non-dipper, *N* (%)	257 (44.5)	212 (35.9)	0.07 (0.01; 0.12)
Masked hypertension, *N* (%)	70 (11.8)	125 (21.0)	−0.09 (−0.13; −0.05)
c-f Pulse wave velocity, m/s*	6.36	6.34	0.02 (−0.04; 0.06)
Basic biochemistry^‡^			
Glucose, mmol/L	4.33	4.91	0.88 (0.86; 0.90)
Total cholesterol, mmol/L	3.72	4.61	0.81 (0.78; 0.83)
HDL-cholesterol, mmol/L	1.26	1.36	−0.10 (−0.15; −0.05)
LDL-cholesterol, mmol/L	2.34	2.98	0.79 (0.74; 0.82)
C-reactive protein, mg/L	1.22	0.98	1.24 (1.03; 1.51)
Family history, *N* (%)			
Hypertension father, yes/unknown	94 (16)/146 (24)	183 (31)/ 111 (19)	−0.15 (−0.20; −0.10)/0.06 (0.01; 0.10)
Hypertension mother, yes/unknown	208 (34)/81 (13)	128 (21)/88 (15)	0.13 (0.08; 0.17)/−0.01 (−0.05; 0.02)
Dyslipidemia father, yes/unknown	12 (2)/194 (32)	183 (31)/124 (21)	−0.28 (−0.33; −0.25)/0.11 (0.06; 0.16)
Dyslipidemia mother, yes/unknown	21 (3)/152 (25)	105 (18)/89 (15)	−0.14 (−0.18; −0.11)/0.10 (0.061; 0.15)
Diabetes father, yes/unknown	56 (9)/102 (17)	61 (10)/31 (5)	−0.01 (−0.04; 0.02)/0.12 (0.08; 0.15)
Diabetes mother, yes/unknown	59 (8)/60 (10)	25 (4)/23 (4)	0.06 (0.03; 0.09)/0.06 (0.03; 0.09)
Current smoker, *N* (%)	154 (25.5)	132 (22.2)	0.03 (−0.02; 0.08)
Current alcohol use, *N* (%)	334 (55.8)	332 (55.8)	−0.01 (−0.06; 0.06)

* Adjusted for clinic mean arterial pressure.

^a^Values are arithmetic mean, or geometric mean for logarithmically transformed variables, or number of participants (%).

^b^Differences between arithmetic means are expressed as absolute values (95% confidence interval; CI) and between geometric means as ratios (95% CI).

^c^At time of publication biochemistry data available for †*N* = 393 black and *N* = 380 white; and ^‡^*N* = 453 black and *N* = 390 white participants.

c-f: carotid-femoral; HDL: high-density lipoprotein; LDL: low-density lipoprotein.

### Ethnic differences

Overall, the clinic and 24-hour BPs, as well as pulse wave velocity were similar between ethnic groups (Table 3), but findings suggest early vascular aging in black participants (reflected by a higher central systolic BP^21^ and an earlier decline in central-to-brachial pulse pressure amplification).^22^ Twenty-four-hour systolic BP was on average 2 mmHg higher in white compared to black participants. Heart rate in black participants was a mean of 2 bpm higher than in white participants. Closer inspection revealed an inadequate nighttime BP dipping^23^ in almost one-half of black (45%) compared to 36% of white participants (Table 3). On the microvascular level we found a smaller central retinal artery diameter in black compared to white participants,^24^ known to reflect an increased risk of hypertension. Regarding biomarkers for endothelial function, young black participants presented with a markedly elevated urinary albumin-to-creatinine ratio, gamma-glutamyl transferase,^25^ C-reactive protein and monocyte chemoattractant protein-1,^21,26^ but a more favourable serum cholesterol profile.^22,26^ Self-reported smoking and alcohol use were also similar between black and white ethnic groups (Table 3).

### Masked hypertension

Approximately one in five white adults (21%) presented at the baseline visit with masked hypertension (i.e. they had normal clinic but elevated out-of-office BP)^27^ (Table 3), compared to a lower prevalence of 12% among black adults. Also, individuals with masked hypertension had an overall increased cardiovascular risk, e.g. increased adiposity, central systolic BP, arterial stiffness and elevated cellular adhesion molecules.^28^ Furthermore, despite their young age those with masked hypertension had a greater adjusted odds ratio (1.67, 95% confidence interval 1.05–2.71, *P* = 0.031) for increased left ventricular mass index.^29^

### Salt intake (24-hour urinary sodium)

The median estimated salt intake of the African-PREDICT study population is 7.9 g/day (5.44; 11.1 g/day, 25th and 75th percentiles; *N* = 773), based on 24-hour urinary sodium. Four out of five exceed the WHO recommendation of less than 5 g/day, and with no ethnic difference in salt intake, but a higher intakes in men than women.^30^ The majority (93%) also did not meet the WHO recommended potassium intake.^30^ Already in this young population, excessive salt intake of 15.6 g/day was found to be positively associated with large artery stiffness, independent of age, sex, BP and other potential confounders, especially in black participants.^31^ In light of findings indicating that salt is stored in the skin, it was found that 24-hour urinary sodium was independently associated with body surface area, but not traditional obesity estimates,^32^ thereby suggesting that additional studies are required on the role of the skin in salt handling. Methodologically, three formulae (Kawasaki, Tanaka and INTERSALT) were evaluated to determine if spot urine can be used instead of 24-hour urine samples to estimate salt intake, using Bland–Altman plots. The results suggest that these formulae should be used with caution, concluding that 24-hour urine collection is still advisable.^33^ To understand sodium handling better, the endogenous steroidal Na/K-ATPase inhibitor, marinobufagenin, was investigated. Similar marinobufagenin levels were found in black and white adults (with higher levels in men), and it was significantly positively associated with urinary sodium. However, marinobufagenin consistently showed independent positive associations with systolic BP (central, daytime, nighttime),^34^ left ventricular mass index^35^ and large artery stiffness^36^ only in women.

### Obesity in young adults

Consistent with several international reports documenting the rise of obesity (defined as body mass index >30 kg/m^2^) in South Africa,^37^ overall, 46% were classified as overweight or obese (26% overweight, 20% obese), despite the young age of the population. Obesity was most prevalent in black women and white men,^32^ confirming previous national reports.^4^ Related to obesity, the adipokine, leptin, was markedly elevated in obese individuals.^26^ Leptin was found to be independently associated with autonomic neural activity, suggesting an early influence of elevated leptin on autonomic function and future BP elevation. This was more evident in men.^26^

## Discussion

The African-PREDICT study tracks the early phases of cardiovascular disease development in young healthy black and white adults. The study involves a bi-ethnic cohort in which a wealth of data unprecedented in hypertension studies, highly unique on the African continent, is collected. The cohort includes 1202 black (*N* = 606) and white (*N* = 596) men and women (aged 20–30 years), recruited from South Africa, from 2013 to 2017. The sample will undergo measurements every 5 years, with the first follow-up from 2018 to 2022, and another 5-year follow-up from 2023 to 2027.

The African-PREDICT study meticulously measures socioeconomic factors, objective methods to assess health behaviours, biomarkers and uses gold standard methods to assess BP and hypertension-related target organ damage. The study is innovative because it measures longitudinally state of the art ‘omics’ and biomarkers proved to predict hypertension and cardiovascular outcomes.

In the South African Demographic and Health Survey of 2016, almost one in two people aged over 15 years were reported to be hypertensive.^4^ However, hypertension is largely preventable. South Africa, like several other developing countries, has been highlighted as experiencing a unique demographic moment to focus on introducing policies that will reduce the future impact of chronic disease, in particular to minimise the increase in cardiovascular diseases.^38^ It may have significantly greater impact if tailored population and individual targeted prevention strategies are employed – especially in young individuals – to detect, prevent and delay hypertension onset earlier. Successful prevention will not only relieve the financial implications of treatment, but more importantly will dramatically improve the quality of life of African populations. The African-PREDICT study has great potential to lead to novel findings to help develop strategies in preventing early hypertension and treating hypertension in Africa. Findings from the study will therefore form the basis of potential scalable intervention studies that may have public health benefit. Such implementation studies will be conducted in close collaboration with the South African Department of Health, having the potential to lead to policy changes.

Strengths include the prospective design, a unique young healthy bi-ethnic population, highly detailed health behaviour measures, an array of traditional and novel biomarkers and cardiovascular profiling with gold standard measures. The study was performed in sub-Saharan Africa under controlled conditions by a trained multidisciplinary team of researchers.

One limitation is that our sample was not purely random. We did not have an a priori defined sampling frame, therefore recruitment was based on individuals who responded to advertisements and invitations. This is a limitation because not every eligible individual had an equal chance to be recruited. Our recruitment strategy was efficient to accommodate strict inclusion criteria and participant screening to ensure a young bi-ethnic clinic normotensive and healthy cohort. Despite targeted efforts to include black and white participants of comparable SES, it remained quite difficult to do so. However, detailed SES data enabled the development of a SES score as a continuous variable, with reasonable variation within each ethnic group.

With final baseline data for the full cohort now available for statistical analysis, and with prospective follow-up on going, more detailed reports comparing ethnic-specific changes in cardiovascular estimates over time are anticipated to be published in forthcoming years. The data are centrally managed by a data manager, using the REDCap system^39^ (research electronic data capture) hosted at the Hypertension in Africa Research Team (HART), North-West University. Potential collaborators are invited to apply to the principal investigator and corresponding author stating brief objectives of their project and analysis plan.

## Conclusions

The status quo, characterised by the under-diagnosis of hypertension, poor control and the immense economic burden associated with the management of hypertension in Africa is not sustainable and undermines the attainment of sustainable development goals.^40^ By collecting modern and cutting edge biomarkers proved to predict hypertension and cardiovascular outcomes, precision public health may have the potential to lead to novel strategies in preventing and treating hypertension in Africa. The unique profile cohort of the African-PREDICT study and the wealth of knowledge gained may therefore prove to be a valuable stepping stone in an effort to achieve the sustainable development goals by identifying and supporting more effective hypertension prevention strategies, especially in Africa.

## Supplemental Material

Supplemental material for The African Prospective study on the Early Detection and Identification of Cardiovascular disease and Hypertension (African-PREDICT): Design, recruitment and initial examination Click here for additional data file. Supplemental Material for The African Prospective study on the Early Detection and Identification of Cardiovascular disease and Hypertension (African-PREDICT): Design, recruitment and initial examination by Aletta E Schutte, Philimon N Gona, Christian Delles, Aletta S Uys, Adele Burger, Catharina MC Mels, Ruan Kruger, Wayne Smith, Carla MT Fourie, Shani Botha, Leandi Lammertyn, Johannes M van Rooyen, Lebo F Gafane-Matemane, Gontse G Mokwatsi, Yolandi Breet, H Salome Kruger, Tertia van Zyl, Marlien Pieters, Lizelle Zandberg, Roan Louw, Sarah J Moss, Itumeleng P Khumalo and Hugo W Huisman in European Journal of Preventive Cardiology
