# Supplementary material for: The African Prospective study on the Early Detection and Identification of Cardiovascular disease and Hypertension (African-PREDICT): Design, recruitment and initial examination
Source: Eur J Prev Cardiol. 2019 Jan 6;26(5):458–70. doi: 10.1177/2047487318822354 (PMC6423686; doi:10.1177/2047487318822354)
Supplement: Supplemental material for The African Prospective study on the Early Detection and Identification of Cardiovascular disease and Hypertension (African-PREDICT): Design, recruitment and initial examination [file Supplemental_Material.pdf]

The African Prospective study on the Early Detection and Identification of Cardiovascular Disease and Hypertension (African-PREDICT): Design, Recruitment and Initial Examination

Supplementary Material

Aletta E Schutte, Philimon N Gona, Christian Delles, Aletta S Uys, Adele Burger, Catharina MC Mels, Ruan Kruger, Wayne Smith, Carla MT Fourie, Shani Botha, Leandi Lammertyn, Johannes M van Rooyen, Lebo F Gafane-Matemane, Gontse G Mokwatsi, Yolandi Breet, H Salome Kruger, Tertia van Zyl, Marlien Pieters, Lizelle Zandberg, Roan Louw, Sarah J Moss, Itumeleng P Khumalo, Hugo W Huisman

METHODS

The African-PREDICT study was designed to longitudinally track and monitor the development of hypertension in healthy black and white individuals, 20-30 years old. African-PREDICT collects modern and high-tech biomarkers proven to predict hypertension and cardiovascular outcome (such as proteomics and metabolomics).

Sample size estimation: The ethnicity/age/sex/socio-economic status (SES) stratified sampling design is a hybrid one with two distinct components. The first one is a cross-sectional design to evaluate and compare the detailed characteristics and cardiovascular profiles of apparently healthy 1200 black and white men and women (aged 20-30 years). The second aspect of the design includes longitudinal monitoring, every 5 years for 10 years of systematic follow-up of the same detailed characteristics of participants. The sample size for the groups was originally set to 200 black and 200 white in each of the three SES categories of the 20-30 year old age group (for a total of 600 black and 600 white participants). Cross-sectional data analysis will focus on frequencies, proportions $\mathbf{p_1}$ and $\mathbf{p_2}$, means, and other basic statistical measures comparing the two ethnicities using two sample methods.

Hypothesis Testing Approach: **Table A** shows exact statistical power of two-sided tests for comparing two binomial proportions $\mathbf{p_1}$ and $\mathbf{p_2}$ with an alpha level of 0.05 and a

sample size of 100 subjects per stratum for estimating proportions ranging from 10% to 50%. Power calculations were performed using a two-group Z test with pooled variance with a two-sided significance level of 0.05, using PASS 11 software. Highlighted cells are underpowered. Cells not highlighted indicate sufficient, i.e., >80% power. Power improves with increasing stratum size. Power also is higher when the distance $p_1 - p_2$ is large, and power is reduced when the distance $p_1 - p_2$ is small. If the prevalence of a condition in white participants is rare (2%, 4% or 6%), there will be adequate power of >80% to detect proportion differences larger than 18%, 16%, and 14%, respectively when N=100. Hypotheses which collapse across strata (e.g., comparing all black to all white participants, N=200 in each group) will have higher power to detect smaller differences in prevalence p_1 and p_2.

<u>Table A</u>: Exact power of two-sided tests for comparing two binomial proportions p_1 and p_2 with an alpha level of 0.05 and a sample size of 100 in each stratum.

**White group, % abnormality, p_2, N=100**		**Black group,% Abnormality p_1, N=100**						
		10%	20%	30%	40%	50%	60%	70%
**Rare**	**2%**	0.69	1.00	1.00	1.00	1.00	1.00	1.00
	**4%**	0.39	0.96	1.00	1.00	1.00	1.00	1.00
	**6%**	0.18	0.86	1.00	1.00	1.00	1.00	1.00
**Medium**	**10%**	-	0.52	0.96	1.00	1.00	1.00	1.00
	**15%**	0.19	0.16	0.73	0.98	1.00	1.00	1.00
	**20%**	0.52	-	0.37	0.88	1.00	1.00	1.00
**High**	**30%**	0.96	0.37	-	0.32	0.83	0.99	1.00
	**40%**	1.00	0.88	0.32	-	0.31	0.83	0.99
	**50%**	1.00	1.00	0.83	0.31	-	0.31	0.83
	**60%**	1.00	1.00	0.99	0.83	0.31	-	0.32

For example, if the prevalence in whites is rare (2%, 4% or 6%), there will be power to detect proportion differences larger than 8%, 16%, and 14%, respectively when N=200. This will of course further increase if all black and white groups (N=600 each) are collapsed. Therefore, there is high confidence that the sample sizes will have sufficient power (>80%) to address the hypotheses. Furthermore, in most instances more

sensitive continuous outcome variables (which yield more statistical power than binary outcome variables) will be used when comparing characteristics, as well as in cross-sectional and longitudinal regression models. Finally, published studies employing proteomics and metabolomics included total sample sizes ranging from N=25,[1] N=49,[2] N=60[3] and N=623[4] reporting significant and useful findings.

Questionnaires (Table 1 main paper): Questionnaires are completed with the help of a research nurse, a trained research assistant, or a trained postgraduate student, and are done one-on-one in the Clinic. Depending on the nature of the questionnaires, some are done in a private room (e.g. psychological questionnaires), whereas others are performed in a quiet area within the Clinic. Dietary 24-hour recall questionnaires are completed by a trained dietician on site, and on two occasions in the following week (including a weekend day). The five-step multiple-pass approach is used in conducting a 24-hour recall.[5] Each fieldworker uses a standardised dietary collection kit containing example pictures, packages, measurement tools and food models. After all three recalls are collected, they are coded according to the South African Medical Research Council's (SAMRC) Food Composition Tables,[6] and the SAMRC's Food Quantities Manual[7] is used to convert household measures to grams. Psychological questionnaires, measuring self-reported indices of psychological distress, stress and coping, and optimal functioning, are completed by a trained psychologist or intern psychologist one-on-one. Several of the questionnaires used have been previously validated in relevant South African populations.[8-12]

Body Composition and Physical Activity (Table 1 main paper): A trained female researcher uses standard procedures as described by the International Society for the Advancement of Kinanthropometry[13] to obtain height (m), weight (kg), and waist, hip and neck circumferences (cm). With the participant in supine position arm-to-leg bio-electrical impedance assessment (BodyStat, model 1500 MDD, Douglas, UK) is applied to assess lean body mass and body fat percentage. The body fat percentage is calculated by the Bodystat software and equation formula, making use of the measured impedance (a function of resistance and reactance), subject sex, age, weight, height, activity level, and optionally waist and hip circumferences, all keyed in before the electrodes are placed. The Bodystat equations are generalized for standard adults. Both subjective and objective habitual physical activity data are collected. A subjective self-report physical

activity questionnaire (Global Physical Activity Questionnaire (GPAQ[14])) is completed by interview. A light-weight combined heart rate and accelerometry device (ActiHeart®, CamNtech Ltd., England, UK) is also fitted to the chest of each participant with two ECG pads. The device records heart rate, inter-beat-interval and habitual physical activity at 60 second epochs for a maximum of 7 consecutive days. Data collected are downloaded and trimmed with the relevant ActiHeart® software. In the absence of a calibration step test, general population equations are applied to determine total energy expenditure, resting and total energy expenditure in kCal per week.

Blood Pressure (Table 1): We measure clinic brachial BP using appropriately sized cuffs and the Dinamap Procare 100 Vital Signs Monitor (GE Medical Systems, Milwaukee, USA), validated by the British and Irish Hypertension Society and the European Society of Hypertension.[15] Participants are not allowed to smoke, exercise or eat at least 30 minutes beforehand. After sitting for 5 minutes, with the arm supported at heart level, a measurement is taken on the left arm. Thereafter BP is taken on the right arm in duplicate with a 5 minute interval. A final measurement is made on the left upper-arm.

Duplicate clinic central systolic BP is taken with the cuff on the right upper-arm (SphygmoCor XCEL, AtCor Medical Pty. Ltd., Sydney, Australia), after 5 minutes of rest with the participant in the supine position. When duplicate readings differ by >3 mmHg, a third measurement is taken.

24-Hour ambulatory blood pressure (and ECG) is taken by placing an appropriate sized cuff to the participant's non-dominant arm (Card(X)plore, Meditech, Budapest, Hungary, validated by the European Society of Hypertension[16]). The device is programmed to take recordings every 30 minutes during the day (06:00 to 22:00) and hourly at night (22:00 and 06:00). Only participants with more than 70% of valid BP measurements, above 20-day measurements and more than 7-night measurements are included. We used Cardio Visions 1.15.2 Personal Edition (Meditech, Budapest, Hungary) software to obtain 24 hour, day and night heart rate, and 24-hour blood pressure and heart rate variability (HRV) measures as previously described.[17,18] The measurement of the 24-hour HRV indices were automatically calculated by the software, and readings taken

from the time, frequency and geometric domains. The time domain analysis included Standard Deviation of Normal to Normal interval (SDNN) which is a representative of overall HRV activity in the time domain. The frequency domain analysis (determined by the fast Fourier transformation) involve low frequency, (a major index of sympathetic cardiac tone but also having a parasympathetic component, LF=0.04–0.15 Hz) and high frequency (a major reflector of the parasympathetic activity, HF=0.15–0.4 Hz) which were measured at the normalized unit. The low frequency-to-high frequency ratio (LF/HF, reflect sympatho-vagal autonomic balance) was also determined, as well as total power, a global determinant of overall autonomic modulation, which is the estimation of the variance of HRV over a certain period of time. We also assessed the geometric domain using the HRV triangular index. It equally represents the entire HRV and is the total number of NN intervals divided by the number of NN intervals in the modal bin of the NN interval histogram.

Target Organ Damage (Table 1):

Large artery stiffness: We assess carotid-femoral pulse wave velocity (PWV, SphygmoCor® XCEL, AtCor Medical Pty. Ltd., Sydney, Australia) with the participants rested in a supine position. Both the femoral and carotid artery waveforms are captured simultaneously by means of an appropriate size femoral cuff placed on the upper right thigh, and carotid artery applanation tonometry. To determine the PWV travel distance, 80% of the distance measured between the arterial points (carotid-to-cuff measured using an infantometer, and femoral-to-cuff via a tape measure) was calculated. PWV was automatically calculated as distance/pulse transit time. Measures were performed in duplicate, and repeated if PWV differed by more than 0.5 m/s.

Carotid wall thickness: B-mode ultrasonography is used to measure carotid intima media thickness (CIMT) on the left and right common carotid artery (General Electric Vivid E9, GE Vingmed Ultrasound A/S, Horten, Norway), by a single medical technologist. Images from at least two optimal angles of the left and right common carotid artery are obtained. A single reader conducted measurements using a semi-automated program, namely the Artery Measurement Systems software (AMS) II v1.139 (Chalmers University of Technology, Gothenburg, Sweden) and Vascular Research Tools 6 (Medical imaging applications, Coralville, Iowa, USA) to also obtain carotid

distensibility from clips. The cross-sectional wall area (CSWA) is calculated to confirm structural and not functional changes in luminal diameter: CSWA = (d/2+ CIMT) 2-d/2)2, where d denotes luminal diameter.

Cardiovascular reactivity: The validated Finometer device[19-21] (FMS, Finapres Measurement Systems, Amsterdam, The Netherlands) was connected by placing an appropriate size finger cuff on the middle finger of the left hand, followed by a return-to-flow systolic calibration to provide an individual-level adjustment of the finger arterial pressure with the brachial pressure. Two stress tests are conducted after resting measures were obtained: cold pressor test (right hand in 4°C ice water for 1 minute); followed by the Stroop test. The Finometer records systolic and diastolic BP and compute the heart rate, stroke volume, total peripheral resistance, and "Windkessel" compliance of the arterial system. Baroreflex sensitivity is also calculated with the xBRS method.[22]

Retinal microvascular calibre and responses to a light flicker stimulus: Retinal photography and dynamic retinal vessel responses to light flicker provocation are measured using the Dynamic Retinal Vessel Analyzer (Imedos Systems GmbH, Jena, Germany) fitted with a Carl Zeiss Fundus camera FF-450^{plus} (Carl Zeiss, Meditec Jena, Germany). No intake of food or fluid is permitted 1 hour prior to the measurement. Prior to the measurement the research nurse determines intraocular pressures (Tonopen Avia, Reichert, Munich, Germany), and if >24 mmHg no further measurements are made. Methods to determine retinal vessel calibres (central retinal artery and vein equivalents (CRAE, CRVE)) were described earlier.[23] For retinal vessel analysis, the camera is set at 30°. Using RVA 4.61 software, a small artery and vein segment are selected in the upper or lower temporal quadrant of the fundus image, between 0.5-2.0 optic disc diameters away from the margin of the optic disc. Vessel responses are recorded over the duration of three flicker cycles, each consisting of a baseline phase (50 seconds), a flicker period (20 seconds) and recovery (80 seconds). The quality of the recordings were assessed using a cumulative scoring method.[24] The measurement lasts a total of 350s. The raw data generated from retinal vessel analysis is exported to a Microsoft Excel template with macros to analyse the data. Variables derived include

parameters describing the dilation, constriction and end of flicker phase of the vessel response to light flicker provocation.[25]

Echocardiography: A standard transthoracic echocardiogram is performed for each participant (GE Vivid E9, GE Vingmed Ultrasound A/S, Horten, Norway).[26] Echocardiography data are analysed using the EchoPAC software (GE, version 10.8.1) to determine measures of left ventricular structure and function. Each participant is scanned in a partial left decubitus position with the head of the examining table modestly elevated. Left ventricular dimensions are measured according to the recommendations of the American Society of Echocardiography, by one specialist clinical technologist.[26,27] A detailed description on derived variables is published (amongst others left ventricular mass index, relative wall thickness, left ventricular systolic and diastolic function).[28]

Electrocardiography: A standard 12-lead ECG (Norav Medical Ltd, PC 1200, v5.030, Israel) is recorded during resting conditions with the participant in a supine position.

Renal function: Serum cystatin-C and creatinine are measured. We calculate the estimated glomerular filtration rate (eGFR) using the Chronic Kidney Disease Epidemiology Formula (CKD-EPI).[29] Urinary albumin-to-creatinine ratio is determined from spot urine, and urinary albumin excretion is determined from 24-hour urine samples.

Biological biomarkers (Table 1; Table 2): An array of biomarkers are investigated to better understand ethnic-specific development of hypertension. Basic and more detailed markers are analysed on site using the Cobas Integra 400plus (e.g. glucose, lipids, liver enzymes, C-reactive protein, 24-hour urinary sodium and potassium) and Cobas e411 (e.g. sex hormones, n-terminal brain natriuretic peptide) auto-analysers (Roche, Basel, Switzerland); individual markers are analysed using enzyme-linked immunosorbent assays (mainly Quantikine R&D systems, Minneapolis, MN, USA); multiplex assays are analysed on site and elsewhere (MILLIPLEX® Luminex xMAP, Luminex 200™; EMD Millipore, Merck, Missouri, USA). RAS-Fingerprint™ (Attoquant Diagnostics, Vienna, Austria) analyses are performed using LC-MS/MS generated multiplex parameters consisting of the precisely quantified concentrations of 10

angiotensin peptide metabolites. Three analytical platforms including NMR spectroscopy, LC-MS/MS and GC-TOF/MS are used to gather metabolomic data (North-West University). For proteomics, capillary electrophoresis-mass spectrometry (CE-MS) technology is used (University of Glasgow). Several specific analyses are performed by international collaborators (**Table 2**).

REFERENCES

1. van Deventer CA, Lindeque JZ, van Rensburg PJ, Malan L, Van der Westhuizen FH, Louw R. Use of metabolomics to elucidate the metabolic perturbation associated with hypertension in a black South African male cohort: the SABPA study. *J Am Soc Hypertens* 2015; **9**(2): 104-14.

2. Nkuipou-Kenfack E, Duranton F, Gayrard N, et al. Assessment of metabolomic and proteomic biomarkers in detection and prognosis of progression of renal function in chronic kidney disease. *PLoS One* 2014; **9**(5): e96955.

3. Brown CE, McCarthy NS, Hughes AD, et al. Urinary proteomic biomarkers to predict cardiovascular events. *Proteomics Clin Appl* 2015; **9**(5-6): 610-7.

4. Delles C, Schiffer E, von Zur MC, et al. Urinary proteomic diagnosis of coronary artery disease: identification and clinical validation in 623 individuals. *J Hypertens* 2010; **28**(11): 2316-22.

5. Steinfeldt L, Anand J, Murayi T. Food reporting patterns in the USDA automated multi-pass method. *Procedia Food Sci* 2013; **2**: 145-56.

6. Wolmarans P, Danster N, Dalton A, Rossouw K, Schönfeldt H. Condensed food composition tables for South Africa. Parrow Valley, Cape Town: Medical Research Council; 2010.

7. Langenhoven M, Conradie P, Wolmarans P, Faber M. MRC food quantities manual. Cape Town: South African Medical Research Council; 1991.

8. Vorster HH, Kruger A, Wentzel-Viljoen E, Kruger HS, Margetts BM. Added sugar intake in South Africa: findings from the Adult Prospective Urban and Rural Epidemiology cohort study. *Am J Clin Nutr* 2014; **99**(6): 1479-86.

9. Kruger HS, Venter CS, Vorster HH, Margetts BM. Physical inactivity is the major determinant of obesity in black women in the North West Province, South Africa: The THUSA study. *Nutrition* 2002; **18**(5): 422-7.

10. Keyes CL, Wissing M, Potgieter JP, Temane M, Kruger A, van Rooy S. Evaluation of the mental health continuum-short form (MHC-SF) in setswana-speaking South Africans. *Clinical psychology & psychotherapy* 2008; **15**(3): 181-92.

11. Malan L, Schutte AE, Malan NT, et al. Specific coping strategies of Africans during urbanization: comparing cardiovascular responses and perception of health data. *Biol Psychol* 2006; **72**(3): 305-10.

12. Malan L, Schutte AE, Malan NT, et al. Coping mechanisms, perception of health and cardiovascular dysfunction in Africans. *Int J Psychophysiol* 2006; **61**(2): 158-66.

13. International Society for the Advancement of Kinanthropometry (ISAK). International standards for anthropometric assessment. Adelaide, 2001.

14. Bull FC, Maslin TS, Armstrong T. Global physical activity questionnaire (GPAQ): nine country reliability and validity study. *Journal of physical activity & health* 2009; **6**(6): 790-804.

15. Medaval Certified Accuracy. Dinamap ProCare 100. https://medaval.ie/device/dinamap-pro-100/ (accessed 26.11.2018).

16. Medaval Certified Accuracy. Meditech Card(X)plore. https://medaval.ie/device/meditech-cardxplore/.

17. Pieterse C, Schutte R, Schutte AE. Autonomic activity and leptin in Africans and whites: the SABPA study. *J Hypertens* 2014; **32**(4): 826-33.

18. Ahiante BO, Smith W, Lammertyn L, Schutte AE. Leptin and its Relation to Autonomic Activity, Endothelial Cell Activation and Blood Pressure in a Young Black and White Population: The African-PREDICT study. *Hormone and metabolic research = Hormon- und Stoffwechselforschung = Hormones et metabolisme* 2018; **50**(3): 257-66.

19. Schutte AE, Huisman HW, van Rooyen JM, Malan NT, Schutte R. Validation of the Finometer device for measurement of blood pressure in black women. *J Hum Hypertens* 2004; **18**(2): 79-84.

20. Imholz BPM, Wieling W, van Montfrans GA, Wesseling KH. Fifteen years experience with finger arterial pressure monitoring: assessment of the technology. *Cardiovasc Res* 1998; **38**(3): 605-16.

21. Guelen I, Westerhof BE, van der Sar GL, et al. Validation of brachial artery pressure reconstruction from finger arterial pressure. *J Hypertens* 2008; **26**(7): 1321-7.

22. Westerhof BE, Gisolf J, Stok WJ, Wesseling KH, Karemaker JM. Time-domain cross-correlation baroreflex sensitivity: performance on the EUROBAVAR data set. *Journal of Hypertension* 2004; **22**(7): 1371-80.

23. Strauss M, Smith W, Schutte AE. Inter-arm Blood Pressure Difference and its Relationship with Retinal Microvascular Calibres in Young Individuals: The African-PREDICT Study. *Heart Lung Circ* 2016; **25**(8): 855-61.

24. Kotliar K, Hauser C, Ortner M, et al. Altered neurovascular coupling as measured by optical imaging: a biomarker for Alzheimer's disease. *Sci Rep* 2017; **7**(1): 12906.

25. Kotliar KE, Lanzl IM, Schmidt-Trucksass A, et al. Dynamic retinal vessel response to flicker in obesity: A methodological approach. *Microvasc Res* 2011; **81**(1): 123-8.

26. Lang RM, Badano LP, Mor-Avi V, et al. Recommendations for cardiac chamber quantification by echocardiography in adults: an update from the American Society of Echocardiography and the European Association of Cardiovascular Imaging. *J Am Soc Echocardiogr* 2015; **28**(1): 1-39.

27. Nagueh SF, Smiseth OA, Appleton CP, et al. Recommendations for the Evaluation of Left Ventricular Diastolic Function by Echocardiography: An Update from the American Society of Echocardiography and the European Association of Cardiovascular Imaging. *European heart journal cardiovascular Imaging* 2016; **17**(12): 1321-60.

28. Sekoba NP, Kruger R, Labuschagne P, Schutte AE. Left ventricular mass independently associates with masked hypertension in young healthy adults: the African-PREDICT study. *J Hypertens* 2018; **36**: 1689-96.

29. Levey AS, Bosch JP, Lewis JB, Greene T, Rogers N, Roth D. A more accurate method to estimate glomerular filtration rate from serum creatinine: a new prediction equation. Modification of Diet in Renal Disease Study Group. *Ann Intern Med* 1999; **130**(6): 461-70.
